# Derrone Targeting the TGF Type 1 Receptor Kinase Improves Bleomycin-Mediated Pulmonary Fibrosis through Inhibition of Smad Signaling Pathway

**DOI:** 10.3390/ijms24087265

**Published:** 2023-04-14

**Authors:** Ilandarage Menu Neelaka Molagoda, Sobarathne Senel Sanjaya, Kyoung Tae Lee, Yung Hyun Choi, Joyce H. Lee, Mi-Hwa Lee, Chang-Hee Kang, Chang-Min Lee, Gi-Young Kim

**Affiliations:** 1 Department of Marine Life Sciences, Jeju National University, Jeju 63243, Republic of Korea; neelakagm2012@gmail.com (I.M.N.M.);; 2 Department of Bioprocess Technology, Rajarata University of Sri Lanka, Mihintale 50300, Sri Lanka; 3 Forest Bioresources Department, Forest Microbiology Division, National Institute of Forest Science, Suwon 16631, Republic of Korea; leekt99@korea.kr; 4 Department of Biochemistry, College of Korean Medicine, Dongeui University, Busan 47227, Republic of Korea; choiyh@deu.ac.kr; 5 Department of Molecular Microbiology and Immunology, Division of Biology and Medicine, Brown University, 185 Meeting St., Providence, RI 02912, USA; joyce_lee1@brown.edu; 6 Nakdonggang National Institute of Biological Resources, Sangju 37242, Republic of Korea; blume96@nnibr.re.kr (M.-H.L.); ckdgml3735@nnibr.re.kr (C.-H.K.)

**Keywords:** derrone, pulmonary fibrosis, TGF-β1, TGF type 1 receptor kinase, Smad2/3

## Abstract

Transforming growth factor-β (TGF-β) has a strong impact on the pathogenesis of pulmonary fibrosis. Therefore, in this study, we investigated whether derrone promotes anti-fibrotic effects on TGF-β1-stimulated MRC-5 lung fibroblast cells and bleomycin-induced lung fibrosis. Long-term treatment with high concentrations of derrone increased the cytotoxicity of MRC-5 cells; however, substantial cell death was not observed at low concentrations of derrone (below 0.05 μg/mL) during a three-day treatment. In addition, derrone significantly decreased the expressions of TGF-β1, fibronectin, elastin, and collagen1α1, and these decreases were accompanied by downregulation of α-SMA expression in TGF-β1-stimulated MRC-5 cells. Severe fibrotic histopathological changes in infiltration, alveolar congestion, and alveolar wall thickness were observed in bleomycin-treated mice; however, derrone supplementation significantly reduced these histological deformations. In addition, intratracheal administration of bleomycin resulted in lung collagen accumulation and high expression of α-SMA and fibrotic genes—including TGF-β1, fibronectin, elastin, and collagen1α1—in the lungs. However, fibrotic severity in intranasal derrone-administrated mice was significantly less than that of bleomycin-administered mice. Molecular docking predicted that derrone potently fits into the ATP-binding pocket of the TGF-β receptor type 1 kinase domain with stronger binding scores than ATP. Additionally, derrone inhibited TGF-β1-induced phosphorylation and nuclear translocations of Smad2/3. Overall, derrone significantly attenuated TGF-β1-stimulated lung inflammation in vitro and bleomycin-induced lung fibrosis in a murine model, indicating that derrone may be a promising candidate for preventing pulmonary fibrosis.

## 1. Introduction

Idiopathic pulmonary fibrosis (IPF) is a prototype of chronic, progressive, and fibrotic lung disease accompanied by many clinical symptoms, including thickening and stiffening of lung tissues and shortness of breath [1]. IPF remains incurable and fatal, due to the destruction of the lung parenchyma with a severe inflammatory response, a lack of promising therapeutics, and a lack of understanding about its precise pathological mechanism [2,3]. IPF mainly occurs in middle-aged and elderly adults and is associated with a histopathological pattern commonly seen in interstitial pneumonia, resulting in respiratory-failure-mediated death [4]. The disease is initiated by excessive infiltration of immune cells into the lung tissue, which subsequently promotes aberrant hyperproliferation of lung fibroblasts, differentiation of lung fibroblasts to contractile myofibroblasts, excessive deposition of the extracellular matrix (ECM), and distortion of normal lung architecture, leading to chronic respiratory failure [5,6]. In summary, the excessive increase in ECM components such as fibronectin, elastin, and collagen destroys the lung parenchyma and the airways, causing IPF.

Transforming growth factor-β (TGF-β) is a fibrogenic cytokine that plays a vital role in the induction and development of pulmonary fibrosis [7,8]. TGF-β-induced acute disturbance of the homeostatic microenvironment promotes cell activation, migration, invasion, and/or hyperplastic changes in the lungs [9,10]. Cheng et al. [11] demonstrated that *TGF-β1* gene silencing significantly downregulated ECM components such as collagen, elastin, and fibronectin in rat liver stellate cells, leading to the treatment of fibrosis, whereas Tarantal et al. [12] found that overexpressing TGF-β1 potentially resulted in severe pulmonary fibrosis in fetal monkeys. Additionally, TGF-β1 initiates the differentiation of fibroblasts into myofibroblasts through a significant increase in α-smooth muscle actin (α-SMA) associated with the acquisition of a contractile phenotype characteristic of myofibroblasts [9,13]. Consequently, myofibroblasts produce primary ECM components, such as fibronectin, elastin, and collagen [14,15]. This indicates that inhibiting the fibrogenic cytokine TGF-β1 and targeting TGF-β signaling pathways are potential strategies for pulmonary fibrosis therapy.

**Figure 1:**
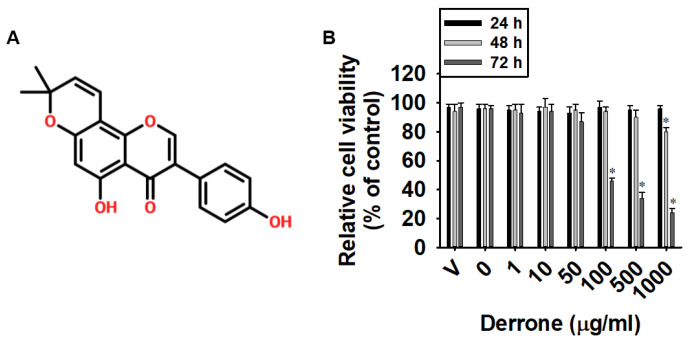
Low concentrations of derrone demonstrate no cytotoxicity in MRC-5 human lung fibroblast. (**A**) Chemical structure of derrone. (**B**) MRC-5 human lung fibroblast cells were seeded at a density of 2 × 10^4^ cells/mL and treated with various concentrations of derrone (0–1000 μg/mL) for 24, 48, and 72 h. Cell viability was measured using an MTT assay. Statistical significance was determined by one-way ANOVA (* *p* < 0.001 vs. untreated cells). V: vehicle control (0.1% EtOH).

*Cudrania tricuspidata* has been widely used as a traditional remedy or health supplement in many Asian countries. Recently, several chemical components have been isolated from *C. tricuspidata*, which have demonstrated neuroprotective, immunomodulatory, and anticancer activities [16,17,18]. Derrone is a prenlylated isoflavone (Figure 1A) from *C. tricuspidata*, and its anti-bacterial and anti-fungal activity is useful against infectious diseases caused by several pathogens [19]. In addition, derrone is believed to be a novel Aurora kinase inhibitor that significantly inhibits the formation and growth of MCF7 tumor spheroids [20]. Kang et al. also demonstrated that derrone induces autophagic cell death in A549 human lung adenocarcinoma cells by increasing cytoplasmic ROS levels and ERK phosphorylation [21]. However, the effect of derrone on IPF remains unknown. Therefore, in this study, we investigated whether derrone suppresses bleomycin-induced pulmonary fibrosis by inhibiting the TGF-β1 signaling pathway.

## 2. Results

### 2.1. Low Concentrations of Derrone Exhibit No Cytotoxicity in MRC-5 Cells

To evaluate the effect of derrone on cell viability, MRC-5 lung fibroblasts were treated with various concentrations of derrone (Figure 1B, 0–1000 μg/mL) or vehicle (V, 0.1% EtOH) for 24, 48, and 72 h and cytotoxicity was assessed using an MTT assay. Derrone did not exhibit any relative cell cytotoxicity during the 24 h treatment (Figure 1B), even at the highest concentration of 1 mg/mL. However, on day 2, derrone induced a slight decrease in cell viability at 1 mg/mL. On day 3, derrone treatment at concentrations from 0.1 to 1 mg/mL resulted in a significant decrease in cell viability (46 ± 3%, 38 ± 5%, and 21 ± 4% at 0.1, 0.5, and 1.0 mg/mL, respectively). Therefore, a derrone concentration of 0.05 mg/mL was chosen for further experiments.

### 2.2. Derrone Downregulates TGF-β1-Induced Myofibroblast Marker Expression

**Figure 2:**
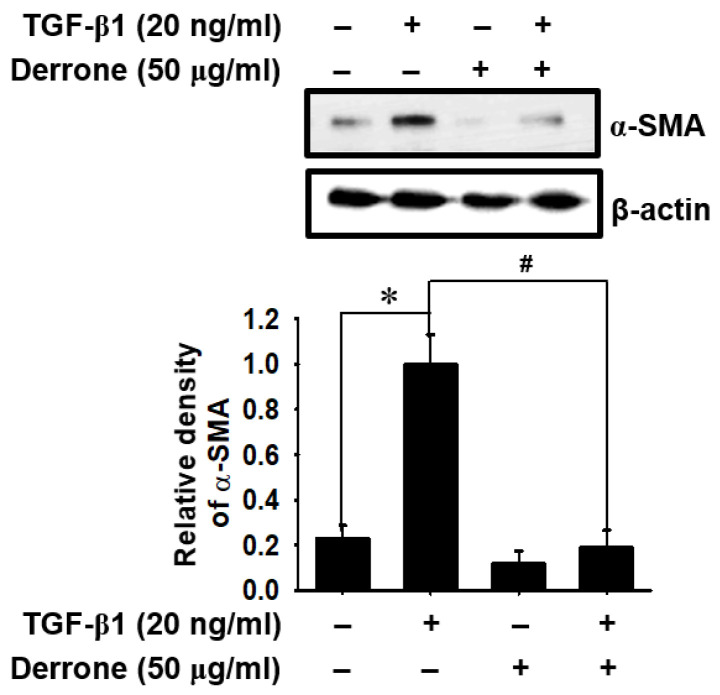
Derrone attenuates TGF-β1-induced α-SMA expression. MRC-5 human lung fibroblast cells were seeded at the density of 5 × 10^4^ cells/mL, and the cells were treated with 50 µg/mL derrone for 2 h followed by 20 ng/mL TGF-β1 for 24 h. Cell lysates were prepared at 24 h, and western blotting was performed using antibodies specific for α-SMA. β-Actin was used as an internal control. Statistical significance was determined via one-way ANOVA and Student’s *t*-test (* *p* < 0.001 vs. untreated cells; *^#^ p* < 0.001 vs. TGF-β1-treated cells). +, treatment and −, untreatment.

**Figure 3:**
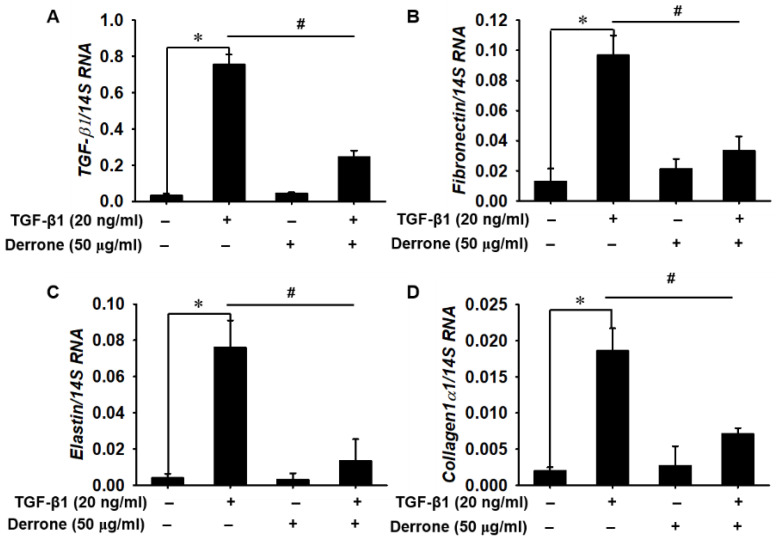
Derrone downregulates the expression of *TGF-β1* and the extracellular matrix (ECM) component genes of *fibronectin*, *elastin*, and *collagen1α1*. MRC-5 lung fibroblast cells were seeded at a density of 5 × 10^4^ cells/mL, and cells were treated with 50 µg/mL derrone for 2 h followed by 20 ng/mL TGF-β1 for 24 h. The relative expressions of (**A**) *TGF-β1*, (**B**) *fibronectin*, (**C**) *elastin*, and (**D**) *collagen 1α1* were measured via quantitative RT-PCR. Statistical significance was determined using the Student’s *t*-test (* *p* < 0.001 vs. untreated cells; *^#^ p* < 0.001 vs. TGF-β1-treated cells). +, treatment and −, untreatment.

To determine whether derrone played an important role in the downregulation of differentiation from lung fibroblasts to myofibroblasts induced by TGF-β1, we evaluated the expression of α-SMA in TGF-β1-treated MRC-5 lung fibroblast cells. Western blot analysis showed that TGF-β1 stimulation increased α-SMA expression to five times higher than that in untreated cells, and the expression was markedly downregulated in the presence of derrone (Figure 2). In addition, we examined the expressions of *TGF-β1* and ECM component genes in MRC-5 cells using quantitative RT-PCR. Our results demonstrated that the TGF-β1 treatment significantly increased the expression of myofibroblast differentiation marker genes, including *TGF-β1* (Figure 3A) and the ECM component genes of *fibronectin* (Figure 3B), *elastin* (Figure 3C), and *collagen1α1* (Figure 3D); these increases reached up to approximately 10 times higher than those in untreated cells. By contrast, treatment of the cells with derrone significantly downregulated the expression of the genes comparable to that of the untreated cells. The results revealed that derrone inhibited TGF-β1-induced myofibroblast differentiation of MRC-5 lung fibroblast cells and downregulated the expression of ECM components.

### 2.3. Derrone Attenuates Pulmonary Fibrosis in Lungs of Bleomycin-Challenged Mice

**Figure 4:**
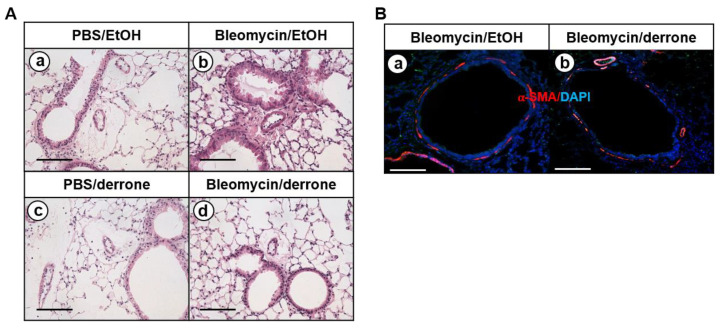
Derrone alleviates bleomycin-induced pulmonary fibrosis in mice. Mice were intratracheally instilled with bleomycin (2.5 U/kg mouse). Derrone (500 µg/kg mouse) was received intranasally every three days following bleomycin instillation. (**A**) The mice were sacrificed on day 14, and lung samples were collected. Paraffin sections from lung tissues of the mice on day 14 were stained with hematoxylin and eosin (H&E) staining. (**a**) Phosphate-buffered saline (PBS)/ethanol (EtOH, 0.1%); (**b**) bleomycin/EtOH; (**c**) PBS/derrone; (**d**) bleomycin/derrone. Scale bar = 100 μm. (**B**) In a parallel experiment, the paraffin sections from lung tissues were stained with α-SMA (red) and DAPI (blue). Bleomycin—EtOH treatment in the absence (**a**) or presence (**b**) of derrone. Scale bar = 20 μm.

To determine whether derrone attenuates bleomycin-induced pulmonary fibrosis in vivo, the fibrotic response was histologically examined following bleomycin treatment for 14 days. H&E staining showed a normal histological appearance, with intact alveoli and normal interstitium in the lungs of PBS- (Figure 4A(a)) and derrone-treated (Figure 4A(c)) mice. In contrast, the lungs from bleomycin-treated mice showed markedly increased features of pulmonary fibrosis, including a high number of interstitial cells, and congestion and remodeling of the alveolar airspaces (Figure 4A(b)) compared to features in PBS-treated mice. Treatment with derrone markedly downregulated alveolar thickness and fibrotic masses induced by bleomycin and maintained significantly fewer fibrotic areas in the lungs (Figure 4A(d)). To further examine whether derrone inhibits myofibroblast differentiation from lung fibroblasts in bleomycin-treated mice, the presence of myofibroblasts was detected using α-SMA immunohistochemistry. As shown in Figure 4B(a), treatment with bleomycin markedly increased α-SMA expression in the lungs, indicating that bleomycin promotes differentiation toward myofibroblasts. However, derrone dramatically inhibited α-SMA expression in the lungs of bleomycin-challenged mice (Figure 4B(b)). The results indicate that derrone potentially inhibits bleomycin-induced pulmonary fibrosis accompanied by a significant decrease in myofibroblast differentiation.

### 2.4. Derrone Decreases Collagen Deposition in the Lungs of Bleomycin-Challenged Mice

**Figure 5:**
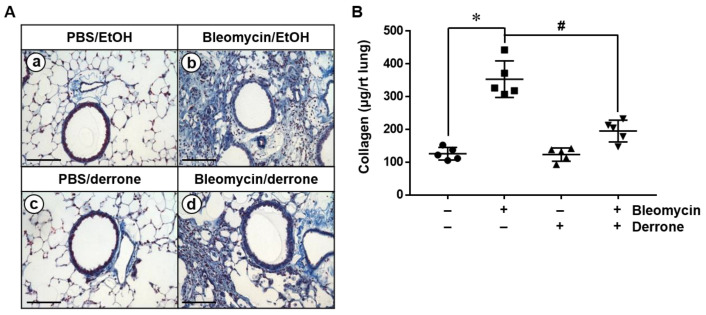
Derrone attenuates collagen deposition in the lungs of bleomycin-challenged mice. Mice were instilled intratracheally with bleomycin (2.5 U/kg mouse). Derrone (500 µg/kg mouse) was received intranasally every three days following bleomycin instillation. The mice were sacrificed on day 14 and lung samples were collected. (**A**) Paraffin sections from lung tissues of the mice on day 14 were stained for collagen deposition, as indicated in blue. (**a**) Phosphate-buffered saline (PBS)/ethanol (EtOH, 0.1%); (**b**) bleomycin/EtOH; (**c**) PBS/derrone; (**d**) bleomycin/derrone. Scare bar = 100 μm. (**B**) Total amount of soluble collagen in right (rt) lung lysates was measured using a Sircol collagen assay. Statistical significance was determined using the Student’s *t*-test (* *p* < 0.001 vs. untreated mice; *^#^ p* < 0.001 vs. TGF-β1-treated mice). +, treatment and −, untreatment.

We performed Masson’s trichrome staining to assess collagen deposition in the lungs of bleomycin-challenged mice. Collagen staining data revealed that PBS- (Figure 5A(a)) and derrone-treated (Figure 5A(c)) mice showed a few collagenous fibers (blue staining). In contrast, bleomycin-challenged mice displayed a large quantity of collagen deposition in the lungs (Figure 5A(b)). However, derrone extensively alleviated collagen deposition in bleomycin-treated lungs (Figure 5A(d)). To further verify our findings, we quantified the total amount of lung collagen. Consistent with histological data, treatment with bleomycin showed a significant increase in collagen deposition in the lungs compared to that in PBS-treated mice (Figure 5B). Bleomycin administration resulted in approximately three times higher lung collagen deposition compared to that in PBS-treated mice, and derrone significantly downregulated the total amount of collagen in the lungs. These results indicate that derrone attenuates collagen deposition in the lungs of bleomycin-challenged mice.

### 2.5. Derrone Attenuates Expression of Fibrotic Markers in Bleomycin-Challenged Mice

**Figure 6:**
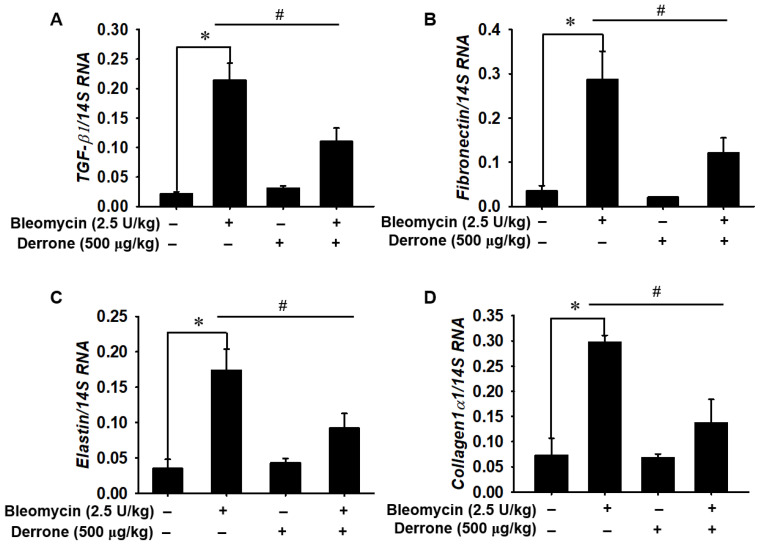
Derrone decreases the expression of *TGF-β1*, *fibronectin*, *elastin,* and *collagen 1α1* in the lungs of bleomycin-treated mice. Mice were intratracheally administrated with bleomycin (2.5 U/kg mouse) at day 0 and derrone (500 μg/kg mouse) was intranasally delivered every 3 days (i.e., days 3, 6, 9, and 12). On day 14, mice were sacrificed, and RNA was isolated from the mouse lungs. Then, the relative expressions of (**A**) *TGF-β1*, (**B**) *fibronectin*, (**C**) *elastin*, and (**D**) *collagen 1α1* were measured using quantitative RT-PCR. Statistical significance was determined using the Student’s *t*-test (* *p* < 0.001 vs. untreated mice; *^#^ p* < 0.001 vs. TGF-β1-treated mice). +, treatment and −, untreatment.

To confirm the anti-fibrotic effect of derrone in bleomycin-treated mice, the expression of ECM genes—including *TGF-β1*, *fibronectin*, *elastin*, and *collagen1α1*—was quantified using real-time RT-PCR. Bleomycin caused a significant increase in the levels of *TGF-β1* (Figure 6A), *fibronectin* (Figure 6B), *elastin* (Figure 6C), and *collagen1α1* (Figure 6D) in murine lung tissues compared to PBS-treated mice. On average, mice treated with bleomycin increased expressed ECM-related genes at levels 10 times higher than those of PBS-treated mice. In contrast, treatment with derrone significantly downregulated the expression of all tested ECM-related genes, with expression levels slightly higher than those in PBS-treated mice. The above data indicate that derrone inhibits the expression of ECM-related genes in bleomycin-induced lung fibrosis.

### 2.6. Molecular Docking Based on SwissDock

**Figure 7:**
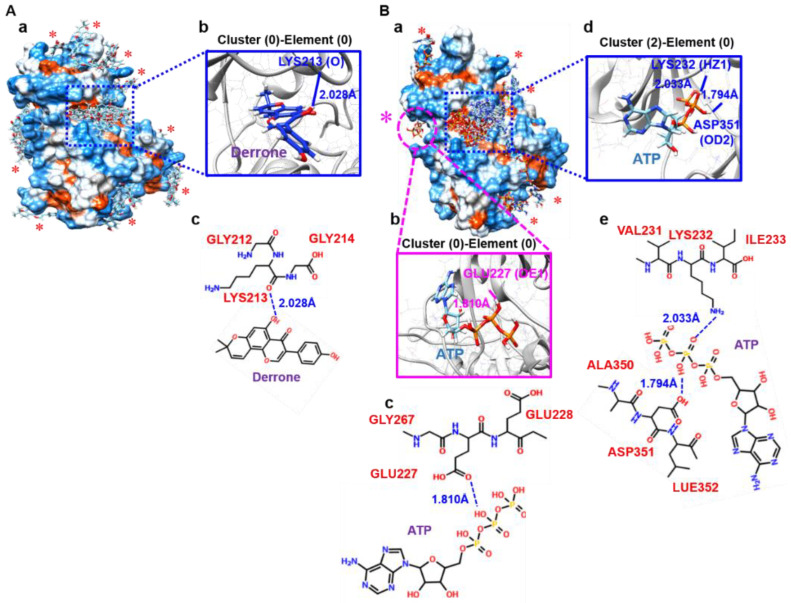
Derrone possibly binds to the ATP-binding site in TGF-β receptor type I (TβRI) kinase domain (ALK5). (**A**(**a**)) Predicable binding sites of derrone (asterisks) to ALK5 (PDB ID: 5E8S) based on SwissDock. The most powerful binding activity [Cluster (0)-Element (0), **A**(**b**)] occurred between derrone and ALK5 at the ATP binding site with LYS231 (O) at 2.028 Å (**A**(**c**)). (**B**(**a**)) Predicable binding sites of derrone (pink dotted circle with a pink asterisk) to ALK5 based on SwissDock. The most powerful binding activity [Cluster (0)-Element (0), pink square, **B**(**b**)] occurred between ATP and ALK5, forming a hydrogen bond with GLU227 (OE1) at 1.810 Å (**B**(**c**)). Most ATP was bound to the familiar A-loop of ALK5 and Cluster (2)-Element (0). (**B**(**d**), blue square) shows the strongest binding activity in the loop, forming hydrogen bonds with LYS232 (HZ1) and ASP351 (OD2) at 2.033 Å and 1.794 Å, respectively (**B**(**e**)).

To identify all possible binding pockets of derrone on an attractive target of ALK5, blind docking was performed using SwissDock for a comparison between derrone and ATP. SwissDock generated all possible binding modes: 31 clusters for derrone and 44 clusters for ATP. As shown in Figure 7A(a), several clusters (0, 4, 11, 14, 17, 20, 24, and 27) intensively bound adjacent A-loops known as an ATP-binding sites with different modes (blue dotted box) were observed, and the most favorable binding of derrone (cluster 0-element 0) also existed in the same pocket. Interestingly, cluster 0-element 0 showed that derrone forms a hydrogen bond with LYS213 (O) at 2.028 Å (Figure 7A(b,c)), and it possessed the lowest fullfitness (−1229.41 kcal/mol) and estimated ΔG (−7.11 kcal/mol). Some minor binding sites were also observed (red asterisks in Figure 7A(a)). To assess whether derrone induces comparative inhibition of ATP, ATP-binding clusters were also predicted using SwissDock. Out of the 44 total clusters, 32 clusters (1–4, 7–9, 11, 13–19, 21, 22, 27, 28, 29, and 32–43) fascinatingly fit into the adjacent A-loop (Figure 7B(a), blue dotted box); this indicates that, as previously known, A-loops represent the most likely binding sites of ATP in ALK5 [22]. Interestingly, the strongest fullfitness (−1229.41 kcal/mol) was found in no predicted ATP-binding site (cluster 0-element 0, a pink dotted circle with a pink asterisk in Figure 7B(a)). The binding built a hydrogen bond at a distance of 1.810 Å between ATP and GLU227 (OE1) in ALK5 (Figure 7B(b,c)). Nevertheless, the strongest estimated ΔG (−10.61 kcal/mol) was observed in cluster 2-elements 3 and 4 in an A-loop of ALK5. Hence, we checked the binding pose of cluster 2 (element 0) and showed that it built two hydrogen bonds at distances of 2.033 Å and 1.794 Å with LYS232 (HZ1) and ASP351 (OD2) in the A-loops of ALK5, respectively (Figure 7B(d,e), respectively). Overall, the binding affinity, including fullfitness and estimated ΔG of derrone to ALK5, was higher than that of ATP, indicating that derrone may more powerfully fit into ALK5 and induce ATP-competitive inhibition.

### 2.7. Molecular Cocking Based on AutoDock Vina

**Figure 8:**
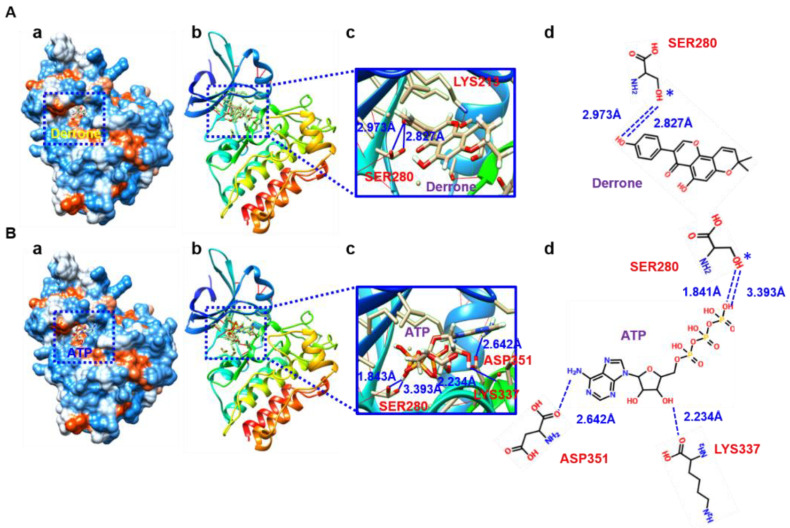
Derrone fits into the ATP-binding site in TGF-β receptor type I (TβRI) kinase domain (ALK5) as shown by AutoDock Vina. The most powerful binding poses (pose 1, blue dotted squares) of derrone (**A**) and ATP (**B**) are shown in the hydrophobicity 3D presentation (**A**(**a**),**B**(**a**)) and ribbon shapes (**A**(**b**),**B**(**b**)). Three-(**A**(**c**),**B**(**c**)) and two-dimensional (**A**(**d**),**B**(**d**)) visualizations of hydrogen bonds and their distances with ALK5.

The docking data from Mcule also showed four strong docking poses of derrone and ATP to ALK5. Four different binding poses were predicted, and, consistent with the SwissDock-based molecular docking data, the docking scores of derrone were generally higher than those of ATP. The strongest binding poses of derrone (Figure 8A(a,b)) and ATP (Figure 8B(a,b)) to ALK5 were bound to the predicted ATP-binding site, A-loop, in depth. Specifically, derrone maintained distances of 2.827 Å and 2.973 Å with SER280 (OG) through hydrogen bonding because 5E8S served two different, alternative positions (blue asterisks, Figure 8A(c,d)). The other three poses confirmed that derrone potently fits in the A-loop. ATP also fits in an A-loop similar to derrone; however, it built four different hydrogen bonds, with SER288 (OG) at 1.841 Å and 3.393 Å owing to alternative positions (blue asterisk), LYS337 (O) at 2.234 Å, and ASP351 (OD1) at 2.642 Å (Figure 8B(c,d)). The other three poses also showed that ATP fits in the A-loop with different binding modes. The above data indicate that derrone is bound to the A-loop of ALK5 and induces competitive inhibition of ATP, thereby leading to the inhibition of the TβRI-mediated signaling pathway.

### 2.8. Derrone Inhibits Phosphorylation and Nuclear Translocation of Smad2/3

**Figure 9:**
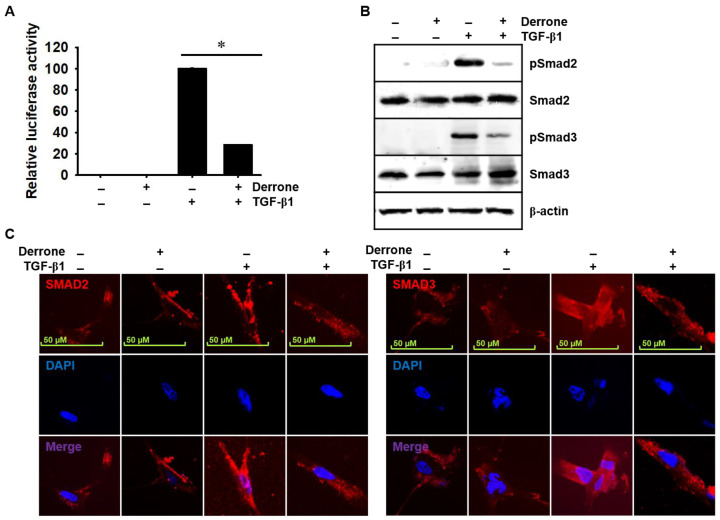
Derrone inhibits TGF-β1-induced SBE-luciferase activity as well as phosphorylation and nuclear translocation of Smad2/3. (**A**) MRC-5 cells were cotransfected with the plasmid SBE-luciferase and *Renilla* luciferase, and derrone (50 μg/mL) was treated 2 h prior to treatment with TGF-β1 (20 ng/mL) for 24 h. Relative luciferase activity is represented as the SEB-luciferase to *Renilla* luciferase ratio. (**B**) MRC-5 cells were treated with derrone (50 μg/mL) for 2 h and stimulated with TGF-β1 (20 ng/mL) for 30 min. Western blotting was performed to detect phospho (p)Smad2 and pSmad3 (red). (**C**) In a parallel experiment, MRC-5 cells were subjected to immunofluorescent staining with anti-Smad2 (**left panel**) and anti-Smad3 (**right panel**). Nuclei were counterstained with DAPI (blue). * *p* < 0.001 vs. untreated mice; +, treatment and −, untreatment.

Because TGF-β1 is one of the most potent activators of lung fibrosis [9,10], we determined that derrone inhibits the TGF-β-dependent Smad signaling pathway using SBE-luciferase activity and phosphorylation of Smad2 and Smad3. The SBE-luciferase reporter gene assay revealed that TGF-β1 significantly increased SBE-luciferase activity in MRC-5 cells; however, derrone dramatically decreased luciferase reporter activity (Figure 9A). Furthermore, TGF-β1 markedly stimulated the phosphorylation of Smad2 and Smad3 (Figure 9B), and, in the presence of derrone, TGF-β1-induced phosphorylation of Smad2 and Smad3 was drastically downregulated. This indicated that derrone possibly binds to the ATP-binding site of ALK5 and consequently prevents the downstream signaling pathway through TGFβR. Additionally, significant increases in the cytosolic and nuclear translocation of Smad2 (Figure 9C, left) and Smad3 (Figure 9C, right panel) were observed in TGF-β1-treated MRC-5 cells; however, expression of TGF-β1-induced Smad2 and Smad3 was decreased and sequestered in the cytosol in the presence of derrone. These data indicate that derrone inhibits TGF-β1-dependent phosphorylation and nuclear translocation of Smad2 and Smad3.

## 3. Discussion

**Figure 10:**
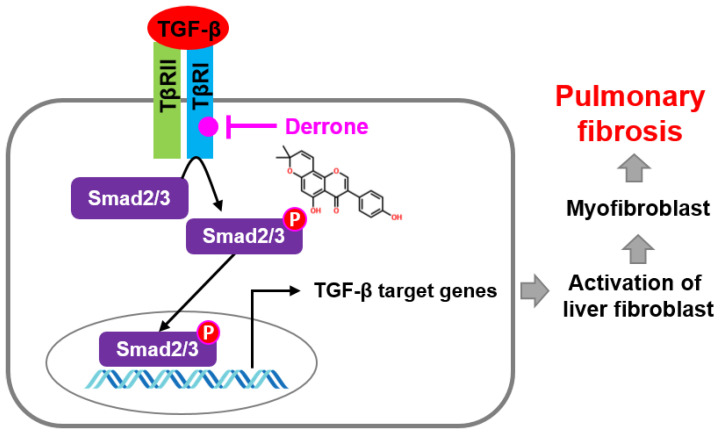
Mechanism of action of derrone. TGF-β, transforming growth factor-β; TβRI, TGF-β receptor I; TβRII, TGF-β receptor II; SMAD, suppressor of mothers against decapentaplegic).

The fruits, bark, and leaves of *C. tricuspidata* have been used in traditional Asian medicine and functional foods to treat many infectious and inflammatory diseases, such as pneumonia, phthisis, paralysis, and hyperpiesia [23]. Recently, many active compounds from *C. tricuspidata*, such as xanthones and isoflavones, were purified and shown to have neuroprotective, antioxidant, anti-cancerous, and anti-inflammatory characteristics [16,17,18]. Derrone is also an active compound isolated from *C. tricuspidata* and inhibits pancreatic lipase, thereby suggesting that derrone may be a beneficial candidate for treating obesity [24]. Additionally, derrone isolated from *Retama raetam* may be a potent antimicrobial agent against pathogens such as *Pseudomonas aeruginosa*, *Escherichia coli*, and the Candida species [19]. Recently, Hoang et al. [20] demonstrated that derrone isolated from *Erythrina orientalis* is an aurora kinase inhibitor that inhibits the phosphorylation of histone H3 at SER10, causing significant inhibition of tumor cell growth and spheroid formation. Nevertheless, whether derrone ameliorates lung fibrosis remains unclear. In this study, we first determined that derrone suppresses bleomycin-induced lung fibrosis, accompanied by a significant decrease in lung fibroblast to myofibroblast differentiation and ECM component expression (Figure 10).

Lung fibrosis is a serious pathological change in the parenchyma accompanied by chronic alveolar epithelial cell injury, myofibroblast hyperplasia, and ECM deposition, which causes respiratory failure [4,25]. The primary results of this process are losses in lung elasticity and alveolar architecture, leading to impairments in gas exchange and pulmonary functions [26]. The bleomycin model of lung fibrosis is the best-characterized and most widely used animal model accompanied by excessive ECM component accumulation [27]. ECM components such as fibronectin, elastin, collagen, and glycoproteins provide a bioactive non-cellular macromolecule network that forms a complex connection between cells and tissues, and regulates cell growth, migration, regulation, and differentiation [28]. However, an excessive accumulation of ECM components in the airway or parenchymal tissues is a marker of many pulmonary diseases, including lung fibrosis [29]. Specifically, aberrant TGF-β1 expression is an early contributor to lung fibrosis by promoting ECM deposition [30]. D’Alessandro-Gabazza et al. [31] reported that a knockdown of TGF-β1 inhibits spontaneous lung fibrosis and improves survival in bleomycin-induced pulmonary fibrosis in mice with a significant decrease in ECM deposition; this indicates that TGF-β1 may be a major factor in inducing fibrosis in the lungs. Recently, dietary supplements and herbal medicines have been shown to prominently slow or prevent lung injury and fibrosis by suppressing TGF-β1-mediated deposition of ECM components [32,33]. In the current study, we evaluated for the first time whether derrone reduces TGF-β1-mediated expression of ECM component genes in MRC-5 lung fibroblasts, differentiation toward myofibroblasts, and bleomycin-induced lung fibrosis in a mouse model. Derrone significantly inhibited the expression of *TGF-β1*, *fibronectin*, *elastin*, and *collagen1α1*, accompanied by a decrease in α-SMA expression in TGF-β1-stimulated MRC-5 lung fibroblast cells. Additionally, intranasal delivery of derrone prevented the expression of ECM component genes and histological alterations, such as increased thickness of the alveolar wall, alveolar congestion, and peribronchial cell infiltration in the lungs of bleomycin-treated mice. The results of this study demonstrated that derrone is a promising candidate for the prevention of lung fibrosis.

TGF-β1 is an important fibrogenic cytokine that promotes fibroblast activation and differentiation to myofibroblasts, and ECM component deposition by binding to the TβRII dimer, thus contributing to airway wall thickness and obstruction [34]. Binding of TGF-β1 to TβRII promotes the recruitment and phosphorylation of the TβRI dimer to form a hetero-tetrameric complex, which canonically stimulates phosphorylation and consequent nuclear translocation of Smad2 and Smad3 [35]. Overall, the TGF-β-mediated Smad2/3 signaling pathway strongly influences the comprehensive pathological action of tissue fibrosis [10,36]. Therefore, the TGF-β-mediated Smad2/3 signaling pathway has been targeted to prevent fibrosis in various tissues [35,36]. In this study, molecular docking data predicted that derrone binds to the ATP-binding site of the TβRI kinase domain with stronger binding activity than ATP, which indicates that derrone interferes with the binding of ATP to the TβRI kinase domain, and consequently inhibits the phosphorylation and nuclear translocation of Smad2 and Smad3. Accordingly, derrone is a potent ATP competitive inhibitor in the structure of the TβRI kinase domain that inhibits tissue fibrosis. Additionally, the signaling pathway is essentially involved in the tumor microenvironment (TME) to increase invasion, metastasis, and immunosuppression by accelerating fibrotic TME concomitant with numerous ECM components [37,38]. From this point of view, derrone is thought to be an excellent inhibitor of the TGF-β-induced TME. As studies on the effects of derrone are insufficient, additional studies on these effects on cancer cell growth should be conducted.

## 4. Materials and Methods

### 4.1. Isolation of Derrone

The leaves of *C*. *tricuspidata* were collected from the Hampyeong Farming Association (Hampyeong, Jeollanam-do, Republic of Korea) and identified by one of the authors (K.T. Lee). A voucher specimen (NIF-11-02-01) was deposited at the Southern Forest Resources Research Center (SFRRC, Jinju, Gyeongsangnam-do, Republic of Korea). Derrone was purified (purity ˃ 99%) following a previously described method [39] and supplied by SFRRC.

### 4.2. Reagents and Antibodies

Human recombinant TGF-β1, 3-(4,5-Dimethyl-2-thiazolyl)-2,5-diphnyl-2H-tetrazolium bromide (MTT), avertin, and 4′6-diamidino-2-phenylinodole dihydrochloride (DAPI) were obtained from Sigma-Aldrich (St. Louis, MO, USA). Bleomycin hydrochloride was purchased from Nippon Kayaku Co., Ltd. (Tokyo, Japan). Antibodies against phospho-Smad2 (pSmad2) (S465/467, #3108, 1:1000) and pSmad3 (S423/425, #9520, 1:1000) from Cell Signaling (Danvers, MA, USA); α-SMA (14-9760-82, 1:1000) from Invitrogen (Carlsbad, CA, USA); and Smad2 (sc-101153, 1:1000), Smad3 (sc-101154), and β-actin (1:1000) from Santa Cruz Biotechnology (Dallas, TX, USA) were purchased. Peroxidase-labeled anti-rabbit (sc-2004, 1:1000) and anti-mouse immunoglobulins (sc-2005, 1:1000) were obtained from Santa Cruz Biotechnology. Alexa Fluor 647-conjugated secondary antibody and Dako Faramount Aqueous Mounting Media were purchased from Abcam (Cambridge, MA, USA) and Dako (Carpinteria, CA, USA), respectively. Dulbecco’s modified Eagle’s medium (DMEM), fetal bovine serum (FBS), and antibiotic mixture were obtained from WELGENE (Daegu, Republic of Korea). All other chemicals were purchased from Sigma-Aldrich.

### 4.3. Cell Culture and Cell Viability Assay

Human MRC-5 lung fibroblasts (American Type Culture Collection, Manassas, VA, USA; ATCC-171) were maintained in DMEM supplemented with 10% FBS and an antibiotic mixture. The cells were maintained at 37 °C in a humidified incubator with 5% CO_2_. Cell viability was determined using an MTT-based in vitro assay. Briefly, MRC-5 cells were seeded in 24-well plates at a density of 2 × 10^4^ cells/well for 16 h and treated with the indicated concentrations of derrone for 24, 48, and 72 h. Following incubation, MTT was added to each well, and the plates were incubated at 37 °C for 4 h. The content of each well was eluted, and the precipitate was dissolved in dimethyl sulfoxide. Absorbance was measured at 540 nm using a microplate reader (BioTek Instruments Inc., Winooski, VT, USA). Relative cell viability (%) was calculated as the ratio of surviving cells to untreated cells.

### 4.4. RNA Isolation and Quantitative Real-Time RT-PCR

The total RNA from MRC-5 cells and lung tissues was isolated using an Easy-Blue Total RNA Extraction Kit (iNtRON Biotechnology, Sungnam, Gyeonggi-do, Republic of Korea) according to the manufacturer’s instructions. RNA extracts were reverse-transcribed using MMLV reverse transcriptase (Bioneer, Daejeon, Republic of Korea). Quantitative real-time PCR (Bio-Rad, Hercules, CA, USA) was used to observe the myofibroblast marker genes. The primers used are listed in Table 1.

### 4.5. Western Blotting 

Cellular proteins were isolated using a PRO-PREP protein extraction solution (iNtRON Biotechnology). Total protein concentrations were measured using the Bradford protein assay kit (Bio-Rad). Equal amounts of protein were loaded and separated onto 10% SDS–PAGE. The resolved proteins were transferred to polyvinylidene difluoride membranes (Amersham, Arlington Heights, IL, USA). The membranes were blocked with 5% skim milk in Tris-buffered saline (pH 7.4) containing 0.1% Tween 20 for 1.5 h at room temperature, followed by overnight incubation with primary antibodies at 4 °C. After washing, the membranes were incubated with horseradish-peroxidase-conjugated secondary antibodies for 2 h at room temperature and protein signals were amplified using an enhanced chemiluminescence reagent (Thermo Fisher Scientific, Waltham, MA, USA).

### 4.6. Murine Model of Bleomycin-Induced Pulmonary Fibrosis

All animal experiments were approved by the Institutional Animal Care and Use Committee of Dongeui University (Busan, Republic of Korea; registration No. A2019-004). All methods were performed in accordance with the ARRIVE guidelines [40]. Eight-week old C57BL/6J mice (KOATECH Laboratory Animals Inc., (Pyeongtaek, Gyeonggi-do, Republic of Korea) were administered a single dose of bleomycin HCl (2.5 U/kg) dissolved in sterilized 0.9% saline through intratracheal administration [5] and randomly divided into four experimental groups (n = 7 in each group): phosphate-buffered saline (PBS, the vehicle of bleomycin) + ethanol (EtOH, the vehicle of derrone), bleomycin + EtOH, PBS + derrone, and bleomycin + derrone. Bleomycin and PBS were intratracheally administered on day 0, and derrone (500 μg/kg mouse) and EtOH were delivered intranasally every four days (on days 3, 6, 9, and 12, specifically). On day 14, the animals were sacrificed using 2.5% 2,2,2-tribromoethanol (Sigma-Aldrich).

### 4.7. Histopathological Analysis

Mice were sacrificed on day 14, and lung samples were collected for histopathological analysis. Briefly, the lungs of the sacrificed mice were collected, immediately fixed with 4% paraformaldehyde (Sigma-Aldrich) for 16 h, and processed using graded alcohol and xylene (Sigma-Aldrich) before being embedded in paraffin wax (Sigma-Aldrich). Tissue sections of 5 μm were collected on microscope slides and stained using a hematoxylin and eosin (H&E) staining kit (ab245880, Abcam) and a Masson’s Trichrome Stain Kit (Polysciences Inc., Warrington, PA, USA).

### 4.8. Immunofluorescence Staining

Tissue sections were then blocked with a non-serum protein-blocking reagent (DakoCytomation Inc., Mississauga, ON, Canada) for 1 h at room temperature and incubated with primary antibodies (1/100 dilution) for 60 min at room temperature in a humid chamber. A substitution of the primary antibody with PBS served as the negative control. The slides were then washed in PBS containing 0.01% Tween 20 and incubated with secondary antibodies conjugated with horseradish peroxidase. Cellular nuclei were stained with DAPI, and the coverslips were mounted onto glass slides with Dako Faramount Aqueous Mounting Media. Fluorescence images were captured using a CELENA S Digital Imaging System (Logos Biosystems, Anyang, Gyeonggi-do, Republic of Korea).

### 4.9. Sircol Collagen Assay

Animals were anesthetized, a median sternotomy was performed, and heart perfusion was completed with calcium- and magnesium-free PBS. The right lung was frozen in liquid nitrogen and stored at −80 °C until use. Collagen contents were determined by quantifying total soluble collagen using a Sircol Collagen Assay Kit (Biocolor, Accurate Chemical & Scientific Co., Ltd. Westbury, NY, USA) according to the manufacturer’s instructions. The data are expressed as the collagen content of the entire right (rt) lung.

### 4.10. Molecular Docking

The TβRI kinase domain (ALK5, PDB: 5E8S) was obtained from the RCSB protein database bank (PDB), and the 3D structures of derrone (PubChem CID: 14704457) and adenosine 5ʹ-triphosphate (ATP, PubChem CID: 5957) were obtained from PubChem (https://pubchem.ncbi.nlm.nih.gov). A molecular docking prediction was performed using SwissDock (http://swissdock.ch, accessed on derrone, swissdockd_2FN0WV_757QD72LRJGOCRRLCVCN; ATP, swissdockd_vg8nix_19BYK97EGP1HV9T6O923) based on EADock DSS [41] and AutoDock Vina-based Mcule (Mcule Inc., Palo Alto, CA, USA, www.mcule.com) [42]. SwissDock served fullfitness (kcal/mol) and estimated ΔG (kcal/mol) on the website. The binding site center in Mcule was 10 Å on the X, Y, and Z axes, and four docking poses were provided. All atoms/bonds were detected within <5 Å from derrone and relax constraints for hydrogen bonds was calculated at 0.4 Å and 20 degrees using USCF Chimera (the Resource for Biocomputing, Visualization, and Informatics at the University of California, San Francisco, CA, USA, www.cgl.ucsf.edu). All other parameters were maintained at their default settings.

### 4.11. Smad-Binding Element (SBE)-Luciferase Assay

MRC-5 cells were subconfluently grown in 24-well plates and co-transfected with 500 ng/mL firefly SEB-luciferase (Addgene, Watertown, MA, USA) and 10 ng/mL pLX313-Renilla luciferase (Addgene) plasmids using Lipofectamine 2000 (Thermo, Waltham, MA, USA). After 24 h of transfection, the cells were treated with derrone (50 μg/mL) for 2 h followed by a TGF-β1 (20 ng/mL) treatment for 24 h. A dual luciferase assay system (Promega, Madison, WI, USA) was used to measure the reporter gene activity. SEB-luciferase activity was normalized to Renilla luciferase activity.

### 4.12. Statistical Analysis

Western blot images were visualized using an ImageQuant LAS 500 (GE Healthcare Bio-Sciences AB, Uppsala, Sweden) and transported to Photoshop. Representative bands were shown in three independent experiments and quantified using the ImageJ bundled with 64-bit Java 8 (https://imagej.net/ij/download.html, accessed on 8 March 2023). Statistical analyses were conducted using SigmaPlot software (version 12.0). Values are presented as the mean ± standard error (SE). Significant differences between the groups were determined using the unpaired Student’s *t*-test and one-way analysis of variance with Bonferroni correction.

## 5. Conclusions

In conclusion, we demonstrated that derrone reduces TGF-β1-induced myofibroblast differentiation by inhibiting the TβRI-mediated Smad2/3 signaling pathway. Administration of derrone in vivo significantly reduced bleomycin-induced lung destruction and ECM component deposition with its regulating genes in mouse lungs. These findings revealed that derrone may be useful in treating lung inflammation and fibrosis. Nevertheless, the specific pathways involved in the development and progression of IPF remain unclear. Therefore, further studies are required to detail the mechanism through which derrone inhibits lung fibrosis.

## Figures and Tables

**Table 1:** Primer sequences used for real-time quantitative PCR.

Species	Gene	Forward Primers (5′→3′)	Reverse Primers (5′→3′)
Mouse	*TGF-β1* *	ttgcttcagctccacagaga	tggttgtagagggcaaggac
	*Fibronectin*	aatggaaaaggggaatggac	ctcggttgtccttcttgctc
	*Elastin*	gctgatcctcttgctcaacc	tccaaacgttcccagaagtc
	*Collagen I α1*	gagcggagagtactggatcg	gcttcttttccttggggttc
Human	*TGF-β1*	cacgtggagctgtaccagaa	gaacccgttgatgtccactt
	*Fibronectin*	cagtgggagacctcgagaag	cactgtgacagcaggagcat
	*Elastin*	ggtggcttaggagtgtctgc	ccagcaaaagctccacctac
	*Collagen I α1*	gtgctaaaggtgccaatggt	ctcctcgctttccttcctct

* *TGF-β, transforming growth factor-β*.

## Data Availability

Data available on request due to restrictions eg privacy or ethical.
